# Efficacy of Non-opioid Analgesics in the Management of Postoperative Pain After Major Abdominal Surgery: A Comprehensive Review and Meta-Analysis of Randomized Controlled Trials

**DOI:** 10.7759/cureus.86157

**Published:** 2025-06-16

**Authors:** Benny Ponappan, Ali Elkandow, Mohammed Gafar Abdelrahim, Mujeeb Ur Rehman, Eman Shaban, Ahmed Shaban, Amira Shaban, Mohammed F Abosamak, Hany A Zaki

**Affiliations:** 1 Emergency Department, Hamad Medical Corporation, Doha, QAT; 2 Cardiology Department, Al Jufairi Diagnosis and Treatment, Doha, QAT; 3 Internal Medicine, Mansoura University Hospitals, Mansoura, EGY; 4 Anaesthesia, Tanta University, Tanta, EGY; 5 Emergency Department, Qatar University - College of Medicine, Doha, QAT

**Keywords:** analgesia, non-opioid analgesics, (nsaid) non-steroidal anti-inflammatory drugs, postoperative pain, surgery, systematic review and meta analysis

## Abstract

Postoperative pain (POP) is one of the leading clinical challenges of patients undergoing major surgeries, including abdominal surgeries. Opioid analgesics are considered the gold standard for POP. However, their use is associated with a high incidence of adverse events, including nausea and vomiting, which has prompted clinicians to look for alternative regimens that are not opioid-based. This review aims to provide an overview of the existing evidence regarding the effectiveness of non-opioid analgesics (NOAs) in the management of POP in patients who have undergone major abdominal surgeries.

A comprehensive search was conducted on four databases: Google Scholar, PubMed, CENTRAL, and Science Direct. The studies that met the inclusion criteria were then included in the review. The reported outcomes were pooled using the Review Manager software (RevMan 5.4, The Cochrane Collaboration, London, UK).

The literature search identified 657 articles, among which 18 were included in the review according to the inclusion criteria. Our study found that the mean postoperative opioid consumption was significantly lower among individuals treated with non-opioid analgesics than with opioid analgesics SMD -1.88; 95% CI (-2.40, -1.36); p < 0.0001). Further analysis showed that the mean opioid consumption was also lower in those who received NSAIDs and other atypical analgesics (SMD -2.24; 95% CI (-2.94, -1.55); p < 0.00001) and (SMD -1.18; 95% CI (-2.18, -0.17); p = 0.02), respectively. However, in those who received paracetamol, the mean opioid consumption was comparable to that of controls (SMD -1.09; 95% CI (-2.21, 0.03); p = 0.06). Secondly, our study found that the incidence of opioid-related nausea was reduced in patients who received NOA than in controls (OR 0.38; 95% CI (0.22, 0.66); p = 0.0005). However, the incidence of vomiting was equivalent across both groups (OR 0.64; 95% CI (0.39, 1.04); p = 0.07).

This study found that NOAs are good adjuvants in pain management in patients undergoing major abdominal surgery. They aid in reducing the dosage of opioids required for adequate analgesia and thus also reduce the incidence of some of the related adverse events.

## Introduction and background

More than 200 million major surgeries are performed worldwide, of which about 20% are major abdominal surgeries [1]. Severe postoperative pain (POP) is one of the significant clinical challenges faced by clinicians and patients. Severe POP has been estimated to occur in about 20 to 40% of all patients who have undergone major surgery [2].

One of the mainstay management approaches for POP is the use of patient-controlled analgesia (PCA) to enable patients to self-administer morphine postoperatively [3]. This analgesic approach is the gold standard for alleviating POP in patients undergoing major surgery [4]. While morphine is regarded as a reference analgesic, it has its limitations, which have prompted clinicians to look for alternative analgesic approaches. These limitations include moderate efficacy in relieving pain when the patient moves and the incapacitating side effects associated with morphine use, such as nausea and vomiting. It may also be prudent to include gastrointestinal manifestations like gastroparesis, reduced bowel motility, and constipation [5].

One of the alternative analgesia strategies is the use of balanced analgesia instead of morphine alone [6]. This strategy, which was proposed more than three decades ago, aims to improve POP management while reducing the use of morphine and its associated side effects. [6] It entails the use of combinations of different analgesics, such as non-steroidal anti-inflammatory drugs (NSAIDs) and other weak opioids, either as monotherapy or in combination with morphine [7].

Different trials have investigated the efficacy of varying monotherapy regimens in managing POP after major surgeries. These trials have investigated the use of monotherapy with or without morphine compared to placebo on POP and postoperative nausea and vomiting (PONV). Furthermore, a summary of these trials has been conducted previously [8,9]. However, none of these meta-analyses focused on POP after major abdominal surgery. This review and meta-analysis, therefore, aim to determine the comparative efficacy of non-opioid analgesics in managing POP after major abdominal surgery.

## Review

Methodology

Protocol and Registration

This review's methodology followed the PRISMA 2020 guidelines (Preferred Reporting Items for Systematic Reviews and Meta-Analyses) [10]. However, the study protocol was not registered in any publicly available database.

Literature Search

Two authors conducted the process independently through a detailed and comprehensive literature search for relevant articles published until October 2024. The search was conducted from the inception of each database until October 2024. Only studies published in English were eligible for inclusion. No restrictions were applied to the publication date or study setting. The search strategy used specified search criteria for each of the four databases, i.e., PubMed, ScienceDirect, CENTRAL, and Google Scholar. Boolean operators ("AND" and "OR") were used to combine relevant keywords and MeSH terms. The full search for PubMed was: (Non-opioid) AND (Analgesics) AND (Acetaminophen OR Paracetamol OR Panadol) AND (NSAIDs OR COX-Inhibitors) AND (Pain OR POP) AND (Major Abdominal surgery). Following the database search, the reviewers manually screened the reference lists from the included studies to identify any additional relevant articles that may have been missed in the electronic search.

Eligibility Criteria

All studies from the three databases were evaluated based on predefined eligibility criteria. Those that fulfilled the inclusion requirements outlined below were selected for the review as described in Table 1.

**Table 1:** Inclusion and Exclusion Criteria for Study Selection

Domain	Inclusion Criteria	Exclusion Criteria
Population	Patients who had undergone major abdominal surgery, as defined by Earl (1917) [11].	Patients who had undergone other types of major surgeries, such as cardiac or orthopedic surgeries.
Intervention	Studies investigating the use of non-opioid analgesics (including nonsteroidal anti-inflammatory drugs, nefopam, acetaminophen, cyclooxygenase-2 inhibitors, and metamizole) in combination with morphine administered solely via patient-controlled analgesia for at least 24 hours.	Studies that did not include non-opioid analgesics as one of the interventions. Studies involving continuous morphine infusion in addition to patient-controlled analgesia were excluded.
Comparison	Trials comparing non-opioid analgesics to placebo, different dosages, or other pharmacological drug classes.	—
Outcomes	Changes in morphine consumption and changes in postoperative pain intensity measured using the visual analog scale.	Studies that did not report any of the outcomes of interest.
Study Design	Randomized controlled trials evaluating non-opioid analgesics used either alone or in combination with other therapies.	Abstracts presented at conferences without full-text articles, single-arm studies, and secondary literature such as systematic reviews or editorial commentaries.

Study Selection and Data Extraction

Study selection was carried out by independent reviewers in multiple phases. Initially, duplicate records were removed. This was followed by a title and abstract screening, and finally, a full-text review of the remaining articles. Abstracts of non-duplicate studies were first assessed against the inclusion criteria. If eligibility could not be determined based on the abstract alone, the full text was retrieved and reviewed.

In addition, the reviewers manually examined results from Phase III clinical trials to identify potentially eligible studies that had not yet been published in peer-reviewed journals.

Once the selection process was completed, the reviewers independently extracted relevant data using standardized, pilot-tested data extraction forms. Data were collected across all reported time points for analysis. Extracted variables included the first author's last name and publication year, the study setting, and the sample size.

Risk of Bias Assessment

The risk of bias across the included randomized trials was evaluated using the revised Cochrane Risk of Bias Tool (RoB 2) [12]. This tool assesses five key domains: the randomization process, deviations from intended interventions, missing outcome data, measurement of outcomes, and selection of the reported results.

Each domain was rated as follows: ‘low risk’ if the criteria were appropriately addressed, ‘some concerns’ if there were issues or uncertainties in the methods, ‘high risk’ if the domain was inadequately addressed, and ‘no information’ when insufficient data were available to make a judgment.

An overall risk of bias rating was determined for each study: ‘low’ if all domains were rated low risk, ‘some concerns’ if one or more domains raised some concerns, and ‘High’ if one or more domains were judged to be at high risk.

Statistical Analysis

Meta-analyses were conducted using Review Manager (RevMan 5.4, The Cochrane Collaboration, London, UK). Continuous outcomes were analyzed using standardized mean differences (SMD), while dichotomous outcomes were analyzed using odds ratios (OR), both with 95% confidence intervals (CI). Heterogeneity was assessed using the I² statistic. Given the anticipated heterogeneity across studies, all analyses were performed using a random-effects model (DerSimonian and Laird method).

Statistical significance was defined as a p-value of ≤ 0.05, and 95% confidence intervals (CIs) were applied to all effect estimates. To assess variability among studies, heterogeneity was evaluated using the I² statistic. Values of I² less than 25% were interpreted as low heterogeneity, 25-50% as moderate, and greater than 50% as high.

Given the anticipated variability across studies, a random-effects model was employed for all meta-analyses.

Results

Search Results

The comprehensive literature search initially identified 663 records across the databases outlined in Table 2.

**Table 2:** Database Search Strategies and Number of Records Retrieved

Database	Search Strategy	Records Retrieved
PubMed	("Non-opioid") AND ("Analgesics") AND ("Acetaminophen" OR "Paracetamol" OR "Panadol") AND ("NSAIDs" OR "COX-Inhibitors") AND ("Pain" OR "POP") AND ("Major Abdominal Surgery")	210
ScienceDirect	("Non-opioid analgesics" AND "postoperative pain" AND "major abdominal surgery")	155
CENTRAL (Cochrane)	("Non-opioid analgesics" AND "postoperative pain" AND "major abdominal surgery")	180
Google Scholar	("Non-opioid analgesics" AND "postoperative pain" AND "major abdominal surgery")	118
Citation Tracking	Manual reference list screening	0

**Figure 1:**
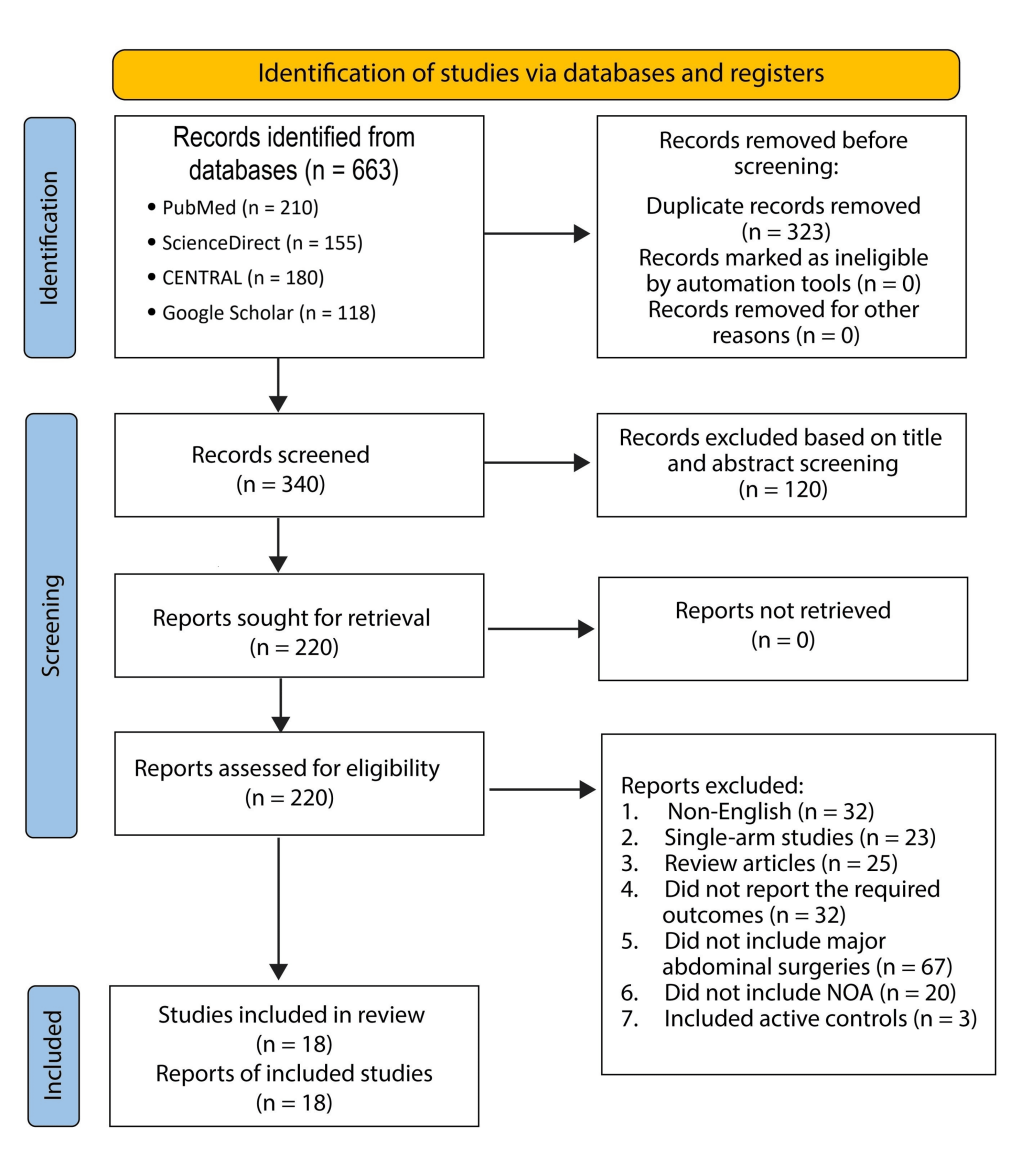
A PRISMA flow diagram summarizing the search strategy

Duplicate records were identified and removed using EndNote 20 reference management software (Clarivate Analytics, Philadelphia, PA), followed by manual verification to ensure completeness. In total, 323 duplicates were excluded. 340 unique abstracts were screened for relevance to the research topic. Of these, 120 were excluded based on their abstract content. The remaining 220 articles were retrieved in full and assessed against the predefined eligibility criteria. Ultimately, only 18 studies met all inclusion criteria and were included in the final review. The reasons for exclusion of the remaining studies are detailed below:22 were not published in English, 23 were single-arm studies, 15 were review articles, 22 did not report the required outcomes, 67 included surgeries other than major abdominal surgeries, 12 did not include non-opioid analgesics as one of their interventions, and eight did not have placebo controls. A PRISMA diagram summarizing the search strategy is outlined in Figure 1.

Characteristics of the Included Studies

This review included 18 randomized controlled trials summarising data from 1894 patients. The RCT was conducted in different settings, including the United Kingdom (UK), the United States of America (USA), Croatia, Australia, South Africa, Egypt, China, Taiwan, Malaysia, Saudi Arabia, Norway, and France. The included patients underwent different procedures, including colorectal surgeries, abdominal hysterectomies, bariatric surgeries, and other major abdominal surgeries. The different NOAs included in the various studies include paracetamol, ketoprofen, ibuprofen, flurbiprofen axetil, naproxen, rofecoxib, ketorolac, lornoxicam, dexmedetomidine, allopurinol, opiranserin, and nefopam. Most of the drugs were given as post-operative continuous doses. On the other hand, a few of the drugs were given pre-operatively as continuous doses, and only one drug was given intraoperatively. The characteristics of the included studies are shown in Table 3.

**Table 3:** The characteristics of the included studies Oberhofer et al., 2005. [13], Rao et al., 2000. [14], Thompson et al., 2000. [15], Celik et al., 2002. [16], Kvalsvik et al., 2003. [17], Chen et al., 2004. [18], Karaman et al., 2006. [19], Bakhamees et al., 2007. [20], Lin et al., 2009. [21], Mowafi et al., 2012. [22], Silinsky et al., 2021. [23], Schmidt et al., 2021. [24], Rindos et al., 2019. [25], Nedeljkovic et al., 2021. [26], Ciftci et al., 2019. [27], Subramaniam et al., 2021. [28], Wang et al., 2020. [29], Raymond et al., 2004. [30] RCT: randomized controlled trial

Author ID	Study Design	Setting	Type of surgery	Analgesic	Sample size	Mean age	Time of administration	Dosage	Type of administration
Oberhofer et al., 2005. [13]	RCT	Croatia	Colon surgeries, gastric surgery, liver resection, and biliary digestive anastomosis.	Ketoprofen	21	66 ± 8.3	Postoperative	100 mg	Continuous
				Placebo	22	63 ± 11.6	Postoperative	0	
Rao et al., 2000 [14]	RCT	Malaysia	Resective bowel surgery	Ketoprofen	20	38.9 ± 6.25	Postoperative	100 mg	Continuous
				Placebo	19	43.1 ± 7.1	Postoperative		
Thompson et al., 2000 [15]	RCT	UK	Abdominal hysterectomy	Meloxicam	18	43.8 (31–70)	Pre-operative	15 mg	Single dose
				Placebo	19	40.9 (30–54)			
Celik et al., 2002 [16]	RCT	Turkey	Abdominal hysterectomy	Naproxen	20	50 ± 9	Pre-operative	550 mg	Single dose
				Rofecoxib	20	52 ± 11		50 mg	
				Placebo	20	52 ± 8			
Kvalsvik et al., 2003 [17]	RCT	Norway	Abdominal hysterectomy	Paracetamol	30	45.0 (39-64)	Postoperative	1 g	Continuous
				Placebo	30	45.0 (35-55)			
Chen et al., 2004 [18]	RCT	Taiwan	colorectal surgery	Morphine + ketorolac	39	64.5 (48.5-71.0)	Postoperative	120 mg plus 100 mg morphine	Continuous
				Morphine	35	68 (47.8-74.0)		100 mg morphine.	
Karaman et al., 2006 [19]	RCT	Turkey	Abdominal hysterectomy	Ketoprofen	20	52.3 ± 12.8	Preoperative	100 mg	Single
				Lornoxicam	20	55.6 ± 11.4		8 mg	
				Placebo	20	53.7 ± 11.9			
Bakhamees et al., 2007 [20]	RCT	Egypt	Laparoscopic gastric bypass.	Dexmedetomidine	40	30 ± 6	Preoperative	0.8 mg/kg	Continuous
				Placebo	40	29 ± 8			
Lin et al., 2009 [21]	RCT	Turkey	Abdominal hysterectomy	Dexmedetomidine	50	43.5 (25–57)	Postoperative	500 μg	Continuous
				Placebo	48	43.5 (25–59)			
Mowafi et al., 2012. [22]	RCT	Saudi Arabia	Abdominal hysterectomy, abdominal myomectomy and radical prostatectomy.	Lornoxicam	20	52.8 ± 16.1	Postoperative	16 mg	Continuous
				Paracetamol	19	49.4 ± 18.4		1 g	
				Placebo	19	48.5 ± 14.4			
Silinsky et al., 2021 [23]	RCT	USA	Laparoscopic or open colorectal surgery.	Meloxicam	26	58.8 ± 11.2	Perioperative	30 mg	Continuous
				Placebo	27	60.6 ± 11.1			
Schmidt et al., 2021 [24]	RCT	Brazil		Allopurinol	27	50.6 ± 1.7	Preoperative	300 mg	Single
				Control	27	49.9 ± 1.7			
Rindos et al., 2019 [25]	RCT	USA	Laparoscopic hysterectomy	Paracetamol	91	41.8 ± 8.3	Perioperative	1 g	Continuous
				Placebo	92	42.1 ± 8.2			
Nedeljkovic et al., 2021 [26]	RCT	USA	Laparoscopic colorectal surgery.	VVZ-149	40	53.9 ± 11.8	Post-operative	1.8 mg/kg loading dose and 1.3mg/kg maintenance dose	Continuous
				Placebo	20	53.9 ± 11.8			
Ciftci et al., 2019 [27]	RCT	Turkey	Laparoscopic sleeve gastrectomy.	Ibuprofen	30	50.16 ± 14.46	Pre-operative	800 mg	Single dose
				Acetaminophen	30	48.10 ± 16.01		1000 mg	
				Placebo	30	43.93 ± 8.58			
Subramaniam et al., 2021 [28]	RCT	USA	Major abdominal surgeries.	Acetaminophen	76	62 (55-71)	Post-operative	1000 mg	Continuous
				Placebo	78	64 (54- 74)			
Wang et al., 2020 [29]	RCT	China	Upper abdominal surgery	Flurbiprofen axetil	118	51.5 (23-75)	Postoperative	100 mg	Continuous
				Tramadol	121	55 (21-75)		100 mg.	
Raymond et al., 2004 [30]	RCT	USA	Lower abdominal surgery	Rofecoxib	16	45.6 ± 11.6	Preoperative	25 mg	Single dose
					16	50.1 ± 13.3		50 mg	
				Placebo	18	48.8 ± 9.8			

Risk of bias

**Figure 2:**
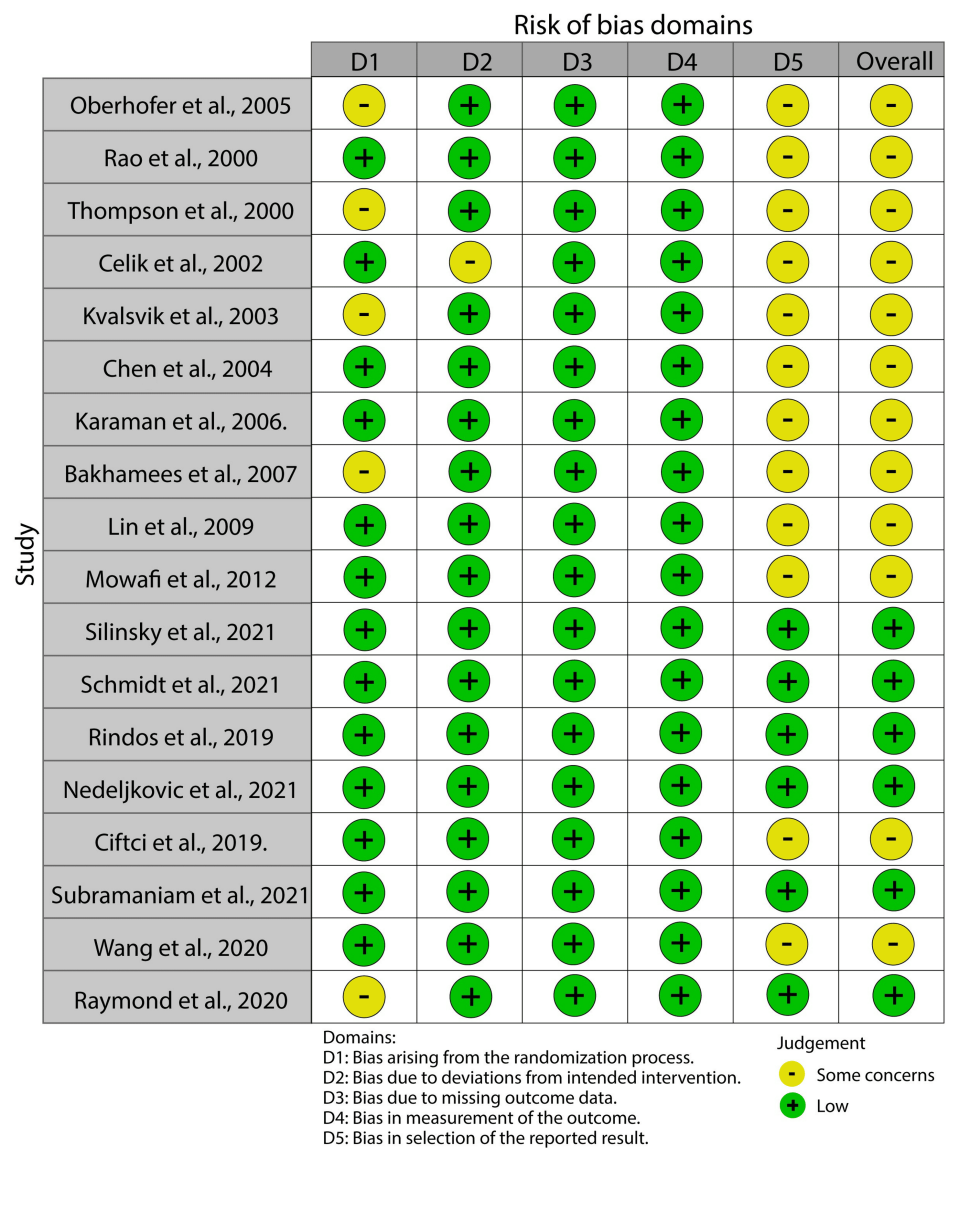
A risk of bias summary showing the risk of bias of the included studies. Oberhofer et al., 2005. [13], Rao et al., 2000. [14], Thompson et al., 2000. [15], Celik et al., 2002. [16], Kvalsvik et al., 2003. [17], Chen et al., 2004. [18], Karaman et al., 2006. [19], Bakhamees et al., 2007. [20], Lin et al., 2009. [21], Mowafi et al., 2012. [22], Silinsky et al., 2021. [23], Schmidt et al., 2021. [24], Rindos et al., 2019. [25], Nedeljkovic et al., 2021. [26], Ciftci et al., 2019. [27], Subramaniam et al., 2021. [28], Wang et al., 2020. [29], Raymond et al., 2004. [30]

The main risk of bias in the included studies was selective reporting bias (Figure 2).

This bias occurred because most of the studies, especially those conducted between 2000 and 2010, did not register the study protocol before its commencement.

Post-operative Opioid Consumption

**Figure 3:**
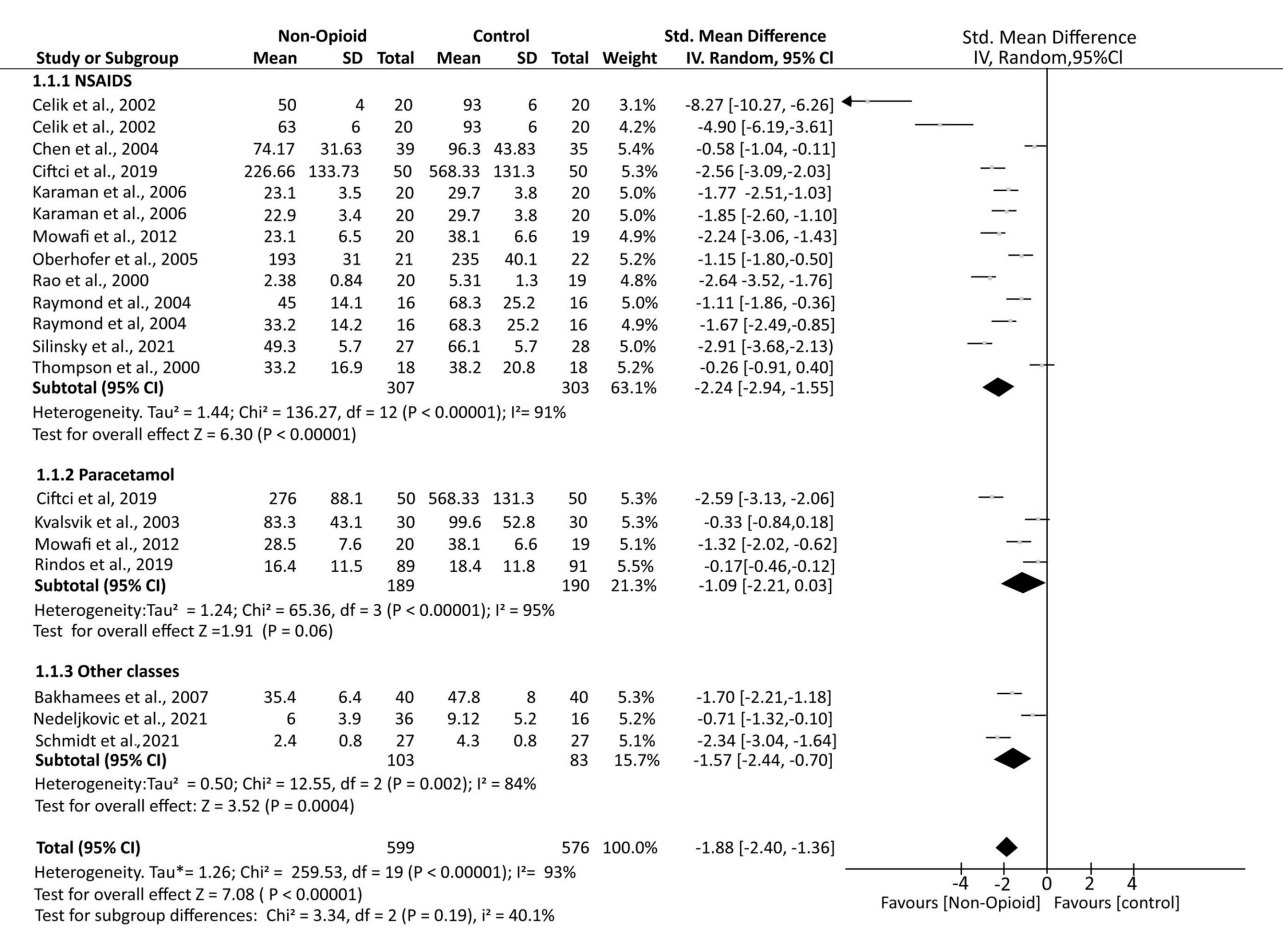
A forest plot showing the mean opioid consumption in the postoperative period Oberhofer et al., 2005 [13], Rao et al., 2000 [14], Thompson et al., 2000 [15], Celik et al., 2002 [16], Kvalsvik et al., 2003 [17], Chen et al., 2004 [18], Karaman et al., 2006 [19], Bakhamees et al., 2007 [20], Lin et al., 2009 [21], Mowafi et al., 2012 [22], Silinsky et al., 2021 [23], Schmidt et al., 2021 [24], Rindos et al., 2019 [25], Nedeljkovic et al., 2021 [26], Ciftci et al., 2019 [27], Subramaniam et al., 2021 [28], Wang et al., 2020 [29], Raymond et al., 2004 [30].

A total of 16 studies reported postoperative opioid consumption. The analysis demonstrated that patients who received non-opioid analgesics (NOA) had significantly lower opioid consumption compared to controls who received a placebo (SMD -1.88; 95% CI (-2.40, -1.36); p < 0.00001). Subgroup analyses showed that patients receiving NSAIDs (SMD -2.24; 95% CI (-2.94, -1.55); p < 0.00001) and other classes of analgesics, including dexmedetomidine, allopurinol, and opiranserin (SMD -1.57; 95% CI (-2.44, -0.70); p = 0.0004), had significantly reduced opioid consumption compared to controls. However, for patients receiving paracetamol, opioid consumption was comparable to controls (SMD -1.09; 95% CI (-2.21, 0.03); p = 0.06). The analysis demonstrated substantial heterogeneity (I² = 93%) (Figure 3).

*Postoperative Pain Scores *(*POP*)

**Figure 4:**
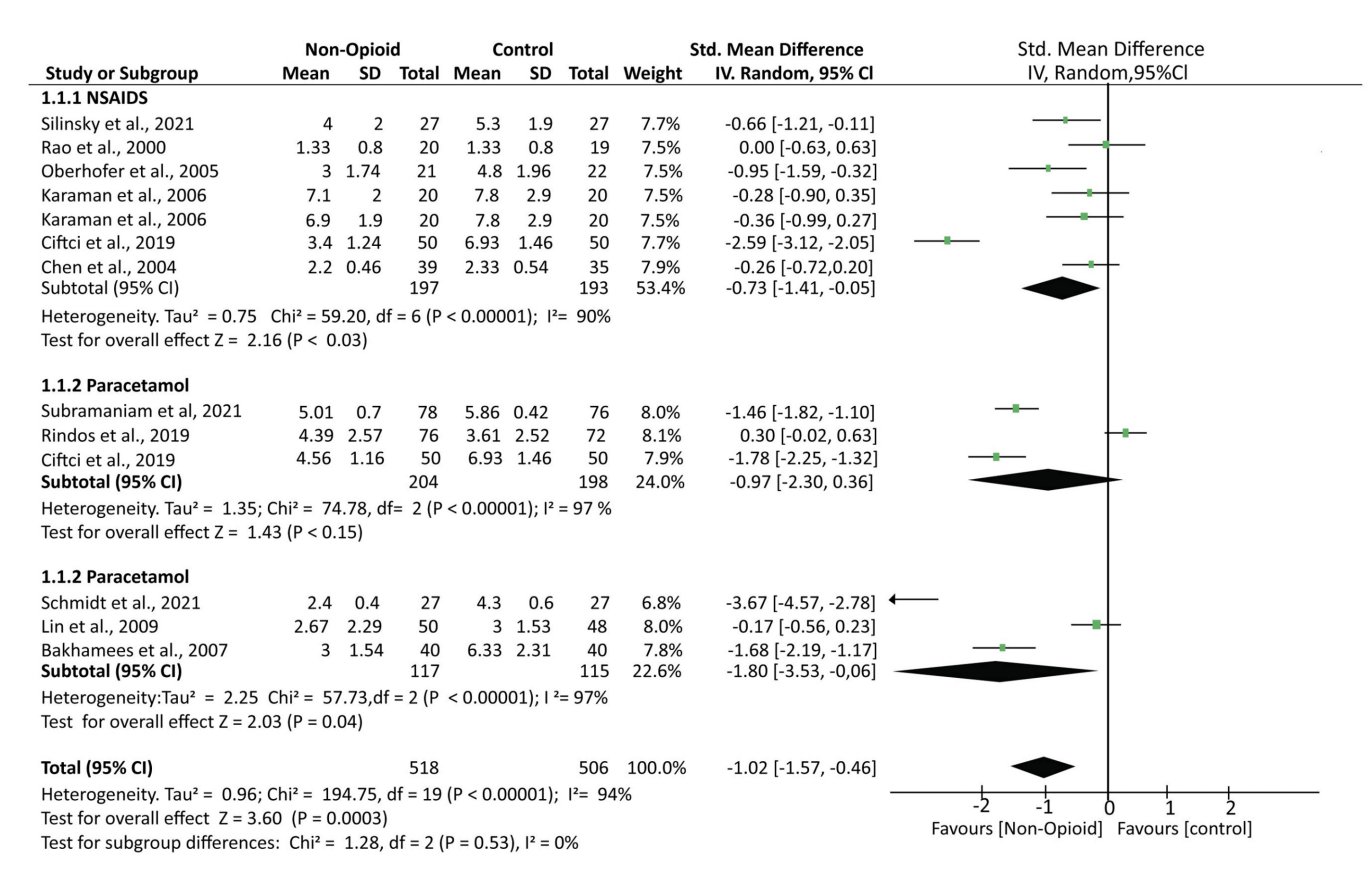
A forest plot showing the mean pain outcomes based on the various pain rating scales Rao et al., 2000 [14], Chen et al., 2004 [18], Karaman et al., 2006 [19], Bakhamees et al., 2007 [20], Lin et al., 2009 [21], Silinsky et al., 2021 [23], Schmidt et al., 2021 [24], Rindos et al., 2019 [25], Ciftci et al., 2019 [27], Subramaniam et al., 2021 [28]

A total of 11 studies reported POP rating outcomes using different scales, including numerical and visual analog scales. A pooled analysis of the reported outcomes showed that the mean pain scores were significantly lower in patients treated with NOA than controls (SMD -1.02; 95% CI (-1.57, -0.46); p = 0.0003). Further subgroup analysis found that the mean pain scores were also significantly lower in patients who received NSAIDs and the other atypical analgesics (SMD -0.73; 95% CI (-1.41, -0.05); p < 0.03) and (SMD -1.80; 95% CI (-3.53, -0.06); p = 0.04). However, the mean pain scores in patients who received paracetamol were comparable to those of controls (SMD -0.97; 95% CI (-2.30, 0.36); p < 0.15). The analysis had high heterogeneity: I² = 94% (Figure 4).

Opioid-Related Adverse Events

**Figure 5:**
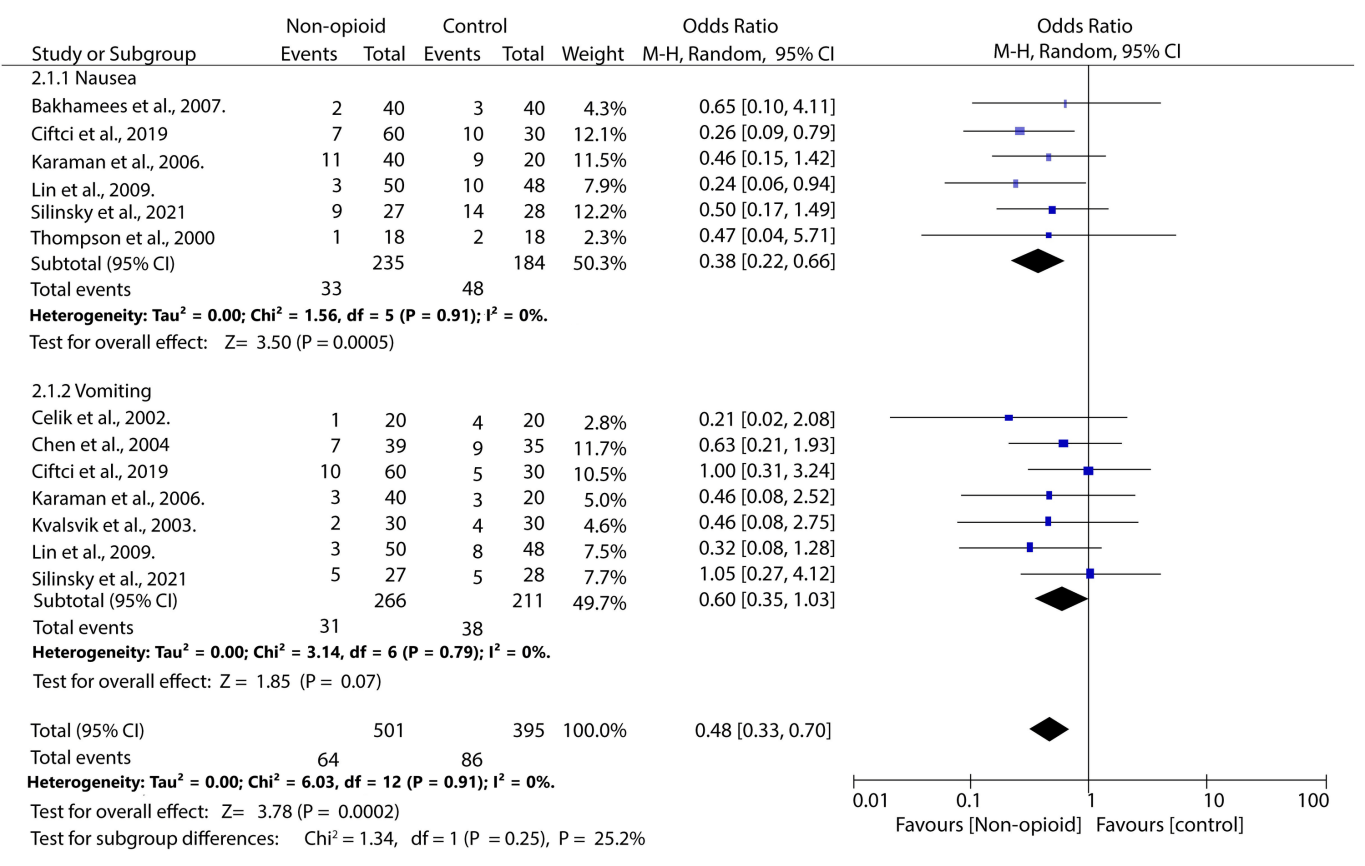
Forest plot illustrating the incidence of adverse events among patients treated with non-opioid analgesics compared to controls. Thompson et al., 2000 [15], Celik et al., 2002 [16], Kvalsvik et al., 2003 [17], Chen et al., 2004 [18], Karaman et al., 2006 [19], Bakhamees et al., 2007 [20], Lin et al., 2009 [21], Silinsky et al., 2021 [23], Ciftci et al., 2019 [27]

We analysed the incidence of two opioid-related adverse events, i.e., nausea and vomiting. Our analysis found that the incidence of nausea was significantly lower in patients who received NOA than in the controls (OR 0.38; 95% CI (0.22, 0.66); p = 0.0005). No significant difference was observed in the incidence of vomiting between patients treated with non-opioid analgesics and those in the control group (OR 0.60; 95% CI (0.35, 1.03); p = 0.07). There was no heterogeneity across the studies (I^2^ = 0%) (Figure 5).

Discussion

Our study found that non-opioid analgesics had superior efficacy to placebo in reducing post-operative opioid consumption in patients who have undergone major abdominal surgeries. Consequently, they provided superior pain relief and also reduced the incidence of opioid-related adverse events in these patients.

Our study analysed the NOA in three groups: NSAIDs, which included traditional NSAIDs such as ibuprofen, and cox-2 inhibitors such as rofecoxib. The second subgroup included studies that investigated the use of acetaminophen. Lastly, the third subgroup included other groups of analgesics, such as dexmedetomidine, an alpha-2 agonist with analgesic effects, and opiranserin, a glycine transporter two agonist. Among the different classes, our study found that NSAIDs had superior efficacy in reducing post-operative opioid consumption. Similar results were found by Martinez et al., who found that NSAIDs had superior efficacy to other analgesics, such as acetaminophen, in managing POP after major surgeries. Martinez et al. further found that combination therapies were even more effective than monotherapies in reducing post-operative opioid consumption and pain scores [5]. However, none of our included studies used combination therapies, and thus, we did not establish the clinical importance of using combination therapies in patients undergoing major abdominal surgeries.

The efficacy of various paracetamol formulations in managing POP has been demonstrated in previous studies [31]. However, our analysis did not show a significant reduction in opioid consumption or POP scores among patients who received paracetamol. In contrast to our findings, a meta-analysis by McDaid et al. reported that paracetamol significantly reduced postoperative opioid consumption following major surgeries [32]. It is, however, essential to note that the efficacy of paracetamol in the meta-analysis was inferior to that of NSAIDs and selective COX inhibitors. These results indicate that paracetamol may not be that efficacious in the management of POP in patients who have undergone major surgeries.

Furthermore, the inferior efficacy of paracetamol in pain management to other analgesics was also demonstrated by Tan et al., who found that ibuprofen was superior in managing pain in children compared to paracetamol [33]. However, the results of our analysis should be interpreted with caution since they are based on a limited number of clinical trials. Further investigation is warranted to establish the efficacy of paracetamol compared to placebo and other analgesics in the management of POP after major abdominal surgeries.

This study also investigated other analgesics, such as dexmedetomidine, an alpha-2 agonist. Dexmedetomidine has been shown to have different beneficial effects in patients who have undergone major surgeries. For instance, a meta-analysis by Poon et al., 2022, found that dexmedetomidine was associated with a reduced mean duration of intensive care and reduced risk of short-term mortality in patients who had undergone cardiac surgery [34]. Furthermore, like our study, a meta-analysis by Zhang et al. found that dexmedetomidine reduced the pain scores of patients and the incidence of postoperative nausea and vomiting in patients who had undergone bariatric surgeries [35]. The last group of drugs investigated in the study is allopurinol, a xanthine oxidase inhibitor mainly used for hyperuricemia [24]. Although not primarily indicated for analgesia, the results of the RCT by Schmidt et al. have shown its efficacy as a preanesthetic analgesic drug [23]. Furthermore, these results indicate that the purinergic system in which allopurinol exerts its mechanism of action is a potential therapeutic target for analgesics [24]. Therefore, further research is warranted to establish its efficacy in analgesia.

The primary aim of introducing NOAs in POP management was due to the associated side effects of opioids that patients face post-operatively [7]. Furthermore, these regimens aimed to reduce the mean opioid consumption and thus reduce the associated side effects [7]. Our study found that using NOAs decreased the incidence of post-operative nausea. However, it is also important to note that caution must be observed since these NOAs also have their associated side effects, which should be closely monitored [35]. For instance, NSAIDs are associated with an increased incidence of bleeding and thus would be a concern in patients, especially after a significant surgical procedure [36]. Fortunately, the analysed studies reported no incidence of postoperative hemorrhage.

Limitations

The main limitation of our study was the high heterogeneity across the studies. The first may be attributed to the different operative procedures the patients in the included studies underwent. Since there is no consensus on the definition of major surgeries, all the surgeries included were of diverse origin from the upper abdomen and the lower abdomen. Furthermore, the operations were either open or minimally invasive laparoscopic surgeries. This diversity in the techniques may have contributed to the heterogeneity observed. Secondly, the reporting of the included outcomes was varied across the studies. For instance, in postoperative opioid consumption, some studies reported the outcomes using the mg while others used milliequivalents; some reported the mean opioid consumption while others reported the cumulative opioid consumption. While the authors used the SMD to enable adjustment of the effect size, this would not adjust for all the variations in the methodology of the different studies.

## Conclusions

The results of this study indicate that non-opioid analgesics (NOAs) are a valuable adjunct to perioperative pain management and contribute significantly to reducing opioid consumption and related adverse events in post-operative patients undergoing major abdominal surgery. Among the evaluated classes, NSAIDs showed the most consistent efficacy, while the role of paracetamol remains less specific. Although dexmedetomidine and other novel agents show promise, further validation is needed.

To further strengthen clinical practice, future studies should focus on head-to-head comparisons of different NOA classes, explore the effectiveness of combination therapies, and investigate patient-specific factors that influence analgesic outcomes. Standardizing outcome measures and surgical classifications across studies will enhance comparability and evidence synthesis. Ultimately, integrating non-opioid analgesics (NOAs) into comprehensive multimodal analgesia protocols-including regional anesthetic techniques such as fascial plane blocks, epidural and intrathecal analgesia, as well as adjuncts like lidocaine and ketamine-may offer a safer and more effective strategy for postoperative pain management in major abdominal surgeries.
